# GABA Levels Are Significantly Reduced in the Visual, Motor, and Auditory Cortex of Patients with Mild Cognitive Impairment

**DOI:** 10.14336/AD.2025.0334

**Published:** 2026-06-26

**Authors:** Mark D. Zuppichini, Abbey M. Hamlin, Quan Zhou, Esther Kim, Kayla Wyatt, Noah Reardon, Benjamin M. Hampstead, Thad A. Polk

**Affiliations:** ^1^Department of Psychology, Montclair State University, Montclair, NJ, USA.; ^2^Departments of Psychology, University of Michigan, Ann Arbor, MI, USA.; ^3^Department of Psychiatry, University of Michigan, Ann Arbor, MI, USA.; ^4^Department of Neurology, University of Michigan, Ann Arbor, MI, USA.; ^5^Department of Psychology, The University of Texas at Austin, Austin, TX, USA.

**Keywords:** GABA, Aging, Mild Cognitive Impairment, MRS, Alzheimer’s Disease

## Abstract

One factor that might contribute to functional deterioration in patients with mild cognitive impairment (pwMCI) is a reduction in the brain’s major inhibitory neurotransmitter, gamma-aminobutyric acid (GABA). Previous studies have reported reductions in GABA in pwMCI while others have not. Here we use magnetic resonance spectroscopy (MRS) to estimate GABA + macromolecules due to co-editing (GABA+) levels in six different brain regions in 37 pwMCI and 163 healthy controls. We estimate GABA+ levels using both creatine and water as reference molecules, and we analyze the effect of correcting for grey matter volume. When referenced to water, we found that GABA+ was significantly lower in pwMCI compared to controls in all six regions, even after tissue composition correction. When referenced to creatine, all but two regions exhibited lower amounts of GABA+ for pwMCI, even after controlling for tissue composition. Results suggest that pwMCI experience reductions in GABA+ throughout the brain, even in regions not typically associated with cognitive impairment.

## INTRODUCTION

Mild cognitive impairment (MCI) is characterized by a decline in function of one or more cognitive domains while other functional capacities remain largely preserved [1]. Roughly 15% of individuals worldwide over the age of 50 years are currently diagnosed with MCI [2], and with an aging population of people over 65 expected to double by 2050, this number is expected to increase. Conversion from MCI to dementia of the Alzheimer’s type (DAT) is common, with one longitudinal study observing a 50-60% conversion rate to DAT from MCI after a 3-year follow-up [3]. Therefore, MCI is often referred to as a transitionary or prodromal phase of DAT that presents as an opportunity to curtail neurodegeneration before it becomes burdensome. To preserve quality of life for patients with MCI (pwMCI), it is critical to understand how and why function starts to decline at this stage.

A unifying theme emerging from literature on MCI/DAT pathology is that synaptic alteration and synaptic loss is strongly correlated with MCI and DAT symptomology [4]. Accordingly, disruption of the neurotransmitter systems receives considerable interest. To date, most research has focused on the cholinergic and glutamatergic systems, but interest in the role of γ-aminobutyric acid (GABA), the brain’s main inhibitory neurotransmitter, in MCI/DAT-related pathology has steadily risen [5]. Research utilizing animal models of Alzheimer’s disease (AD) have found a causal relationship between apolipoprotein E4 (a known genetic risk factor for AD), impaired GABAergic function, and learning and memory deficits [6].

In humans, GABA can be measured *in vivo* via magnetic resonance spectroscopy (MRS). MRS takes advantage of unique molecular resonances and spectral patterns to classify and measure the quantities of various molecules [7, 8]. GABA measured via MRS is utilized to characterize the role of GABAergic dysfunction in various clinical populations, such as schizophrenia [9], multiple sclerosis [10], major depressive disorder [11], autism [12], and substance use disorder [13].

Thus far, studies utilizing MRS to investigate MCI-related differences in GABA have examined GABA concentrations in brain regions typically associated with cognition and MCI-related cognitive impairment and have reported mixed results. For example, Huang, et al. ^(14)^ measured GABA+ referenced to creatine in the right hippocampus and anterior cingulate cortex (ACC) and reported that pwMCI did not exhibit significantly different GABA+/Cr concentrations compared to a healthy control group or AD group. Conversely, Riese, et al. ^(15)^ observed lower GABA+/water levels in the posterior cingulate cortex (PCC) of pwMCI. This result was replicated by Fu, et al. ^(16)^ reporting significantly lower GABA+/Cr and at 7T without spectral editing reporting significantly lower GABA/tCr by Oeltzschner, et al. ^(17)^ in the ACC and PCC of pwMCI.

There are at least two issues that could contribute to the discrepancies between studies. The first is that most of these studies used creatine as a reference when estimating GABA levels, and it is currently unknown whether creatine levels are affected by MCI. If they are, then it could be biased and potentially obscure GABA differences in pwMCI. Specifically, if both GABA and creatine levels are reduced in MCI, and GABA is estimated relative to creatine, then reductions in GABA might be missed. Furthermore, if GABA levels remain stable but creatine levels change, this could mean higher estimates of GABA if creatine declines, or lower estimates of GABA if creatine increases.

A second issue concerns differences in tissue composition and whether these differences are corrected or not. Research has consistently shown that brain volume declines in pwMCI [18, 19]. The rates of atrophy differ between tissue type (i.e., gray or white matter) and vary regionally, with temporal, parietal, and limbic atrophy occurring more rapidly compared to occipital or frontal regions [20-22]. Furthermore, the distribution of GABA across different tissue types is not uniform, with roughly twice as much GABA observed in gray compared to white matter [23, 24]. Discrepancies between studies of GABA levels in pwMCI could therefore be due to differences in tissue composition rather than the differences in GABA *per se*. For instance, in a study of healthy young and old participants, Maes, et al. ^(23)^ reported older participants with lower GABA levels compared to younger participants before tissue correction. However, when GABA measurements were corrected for tissue composition, the age-related differences were no longer observed [23], suggesting that differences were due to tissue composition.

To date, MRS studies in pwMCI have understandably focused on estimating GABA in regions previously associated with MCI deficits (e.g., hippocampal, ACC/PCC). As a result, it is not known whether GABA levels are significantly different in sensory regions, including the auditory cortex, sensorimotor cortex, and ventrovisual areas of pwMCI. Previous research has reported that pwMCI experience auditory processing issues [25], sensorimotor functional decline [26], and fusiform gyrus atrophy associated with cognition [27]. However, it is not known whether GABA levels decline in these areas.

The current study aims to add to our understanding of how the GABAergic neurotransmitter system is affected in pwMCI by estimating GABA+ levels in cortical regions that are not as strongly researched in the context of pwMCI. Furthermore, this study will examine MRS reference choice and tissue correction to assess the robustness of results to these methodological choices.

## MATERIALS AND METHODS

### Participants

All procedures were approved by the University of Michigan Institutional Review Board and all participants provided written, informed consent. Thirty-seven pwMCI (*n* = 37*;* Table 1) and 163 healthy controls (Table 1) were recruited from Ann Arbor, Michigan, and the surrounding area as a part of a larger study. All participants completed the uniform dataset (UDS) v3.0 neuropsychological testing protocol [28]. A board-certified clinical neuropsychologist reviewed test results and available medical information to make a diagnosis of either cognitively intact or MCI using the criteria set forth by the National Alzheimer’s Coordinating Center [28, 29]. Specifically, pwMCI were required to report subjective cognitive change, demonstrate objective cognitive impairment, and be largely independent in instrumental activities of daily living. Twenty-seven pwMCI were classified as amnestic-MCI, and 10 were classified as non-amnestic-MCI. Participants were also screened for MR contraindications and were excluded if pregnant or had any history of neurological or psychiatric disorders, or any history of drug or alcohol abuse.

**Table 1:** Participant Demographics.

Participants	HC (163)	MCI (37)	*p-*value
**Age (years)**	70.5 (5.01)	73.9 (5.21)	.053
**Female/Male**	98/65	19/18	.343
**Education (years)**	16.8 (2.10)	16.0 (2.40)	.063
**MoCA**	26.9 (2.01)	23.0 (4.16)	<.0001

*Note.* Means (standard deviations). Sex was tested using a Chi-square test, all other demographics were tested using *t*-tests.

### MRS data acquisition

**Figure 1.**
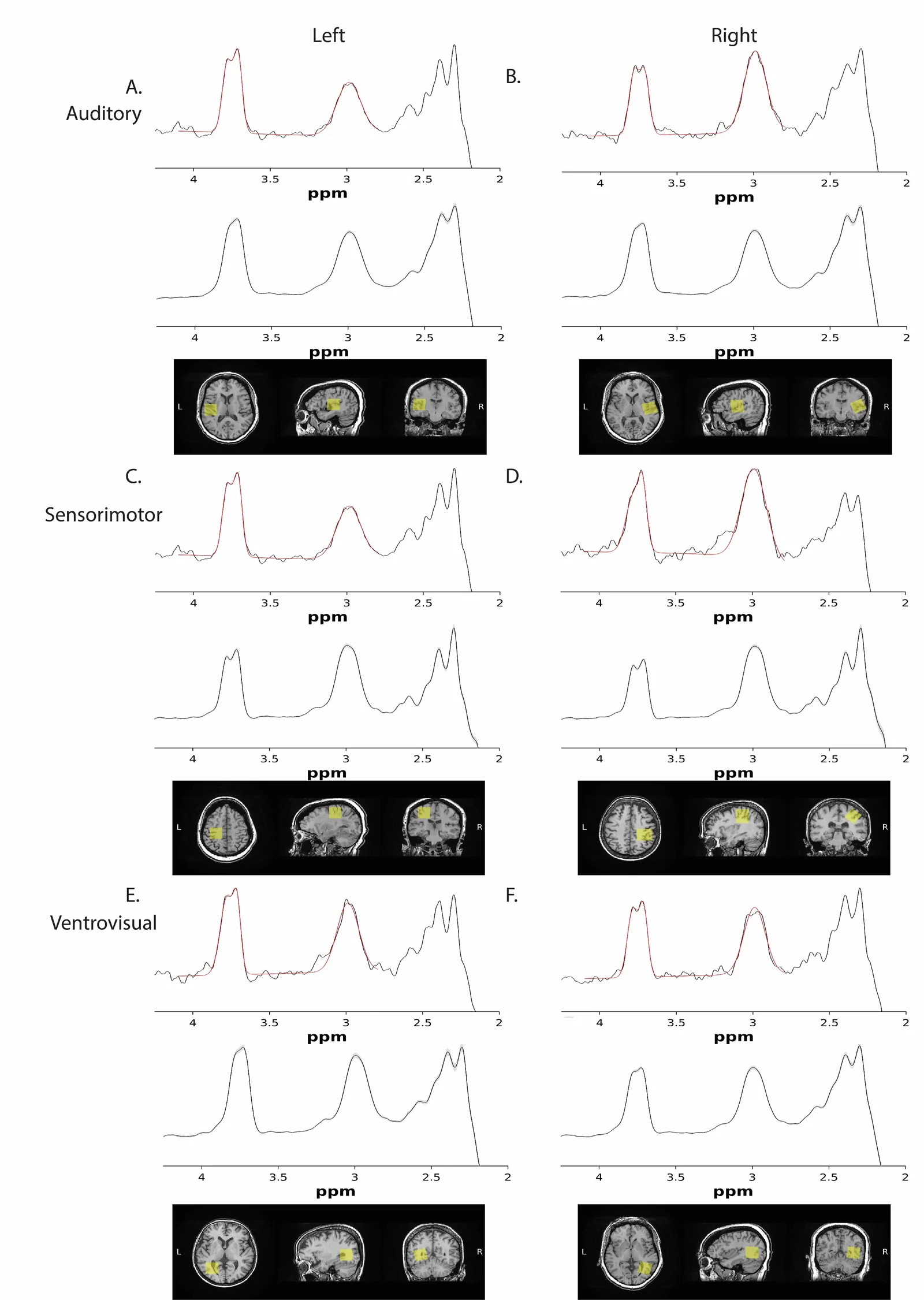
**MRS Spectra and VOIs**. Representative spectrum and model fit (top), average difference spectrum with standard error (middle) and representative voxel placement (bottom) for left **(A)** and right **(B)** auditory voxels, left **(C)** and right **(D)** sensorimotor voxels, and left **(E)** and right **(F)** ventrovisual voxels.

GABA-edited MR spectra were collected from a 3T GE Discovery MR750 scanner with an 8-channel head coil located at the University of Michigan Functional Magnetic Resonance Imaging Laboratory using a MEGA-PRESS sequence [30, 31] with the following parameters: TR = 1800 ms; TE = 68 ms (TE1 = 15 ms, TE2 = 53 ms); 256 transients (128 ON interleaved with 128 OFF) of 4096 data points; spectral width = 5 kHz; frequency selective editing pulses (14 ms) applied at 1.9 ppm (ON) and 7.46 ppm (OFF); FOV = 240 × 240 mm^2^; voxel size = 30 × 30 × 30 mm^3^. Acquisition time for each voxel was ~8.5 min. MRS data were collected from placed in the left and right auditory cortex (Fig. 1A), left and right sensorimotor cortex (Fig. 1B) and, left and right ventrovisual cortex (Fig. 1C). Additionally, a high-resolution T1-weighted spoiled 3D gradient-echo acquisition (SPGR) image was collected for MRS voxel placement and segmentation with the following parameters: Inversion Time = 500ms, flip angle = 15°, field of view = 256 x 256 mm^2^.

To determine voxel placements, a general linear model (GLM) was performed on three functional MRI tasks that were conducted as part of the larger Michigan Neural Distinctiveness study [32], contrasting each experimental condition against rest. Specifically, in a visual task, we computed contrast maps for houses vs. fixation and for faces vs. fixation. Using these two contrast maps and the T1 structural image, we placed the ventral visual voxels to capture the areas of the average highest activation (highest beta value) for the house and face areas in each hemisphere. For the auditory voxels we used contrast maps for speech vs. no sound and music vs. no sound. For the sensorimotor voxels, we placed the left hemisphere voxel to capture activations from a right-hand motor task and the right hemisphere voxel to capture activations from a left-hand motor task. More details about the functional MRI tasks can be found in Gagnon et al. (2019). Voxel placement with fMRI was conducted for each participant and on a separate session from their MRS session so as to not contaminate MRS data quality.

### Quantification of GABA

To estimate GABA levels, we utilized the Gannet 3.3.2 MATLAB toolbox [33]. Time domain data were corrected for phase and frequency using spectral registration [34], filtered with 3 Hz exponential line broadening, and zero-filled by a factor of 16. A Gaussian-Lorentzian model was then fit to the 3-ppm peak in the difference spectrum and GABA levels were quantified relative to both water and creatine. Due to the GABA-editing process, significant excitation of extraneous macromolecules has been reported to contribute to the edited GABA signal at 3ppm [35]. Thus, when referring to GABA, we will use GABA+ to represent GABA plus macromolecules.

### MRS Data Quality

As a measure of quality of fit, Gannet calculates a measure of model fit error that equals the ratio of the standard deviation of the fit residual normalized by the amplitude of the fitted peak. Because GABA was referenced to water and creatine, Gannet documentation (https://markmikkelsen.github.io/Gannet-docs/data-quality-metrics.html#Fit_error) recommends combining the fit errors for GABA and water, and also for GABA and creatine. Those values, along with linewidth and signal-to-noise (SNR) ratios are presented in Table 2 for MCI and healthy controls below. To estimate the degree of scanner-related frequency drift and participant motion, the average frequency offset ( $\overset{®}{\Delta \delta_{0}}$) was calculated as the mean difference between the observed frequency of the residual water signal in the pre-frequency-corrected sub spectra and the nominal water frequency at 4.68 ppm. Values of $\overset{®}{\Delta \delta_{0}}$for each voxel are shown in Table 2. A study-averaged difference spectrum, representative voxel placement and a representative spectrum with model fit are presented in Figure 1.

### Tissue corrections

The main aim of this study is to compare GABA levels in pwMCI and healthy controls while accounting for the effect that differences in tissue composition may have on the interpretation of results. The primary goal of correction is to remove the effect that tissue composition has on the measurements of GABA+. We therefore performed a group α-tissue correction as proposed by Harris et al. (2015). This correction attempts to account for the different relaxation constants and water visibility in different tissue types as well as the expected ratio of GABA in gray matter vs. white matter (2:1). To make GABA+ measurements comparable between groups of interest, this correction method goes further to normalize GABA+ measurements by a standard voxel composition created from each group of interest. Segmentation and co-registration of the T1-weighted anatomical image was performed using the SPM12 segmentation function that is integrated into Gannet 3.3.2 [36].

**Table 2:** MCI and HC Data Quality Metrics for GABA Estimates.

MCI Participants					
**VOI**	**Fit Error, H2O (%)**	**Fit Error, Cr (%)**	**Linewidth (FWHM; Hz)**	**SNR**	**Δδ_0_ (ppm)**
**LAUD**	4.23 (1.24)	4.42 (1.24)	19.62 (2.26)	11.21 (2.41)	0.01 (0.01)
**RAUD**	4.51 (1.35)	4.65 (1.31)	19.40 (1.77)	11.47 (2.58)	0.004 (0.02)
**LSM**	4.19 (0.98)	4.43 (0.95)	17.62 (1.19)	12.57 (2.95)	0.001 (0.01)
**RSM**	4.11 (0.69)	4.33 (0.66)	17.68 (1.30)	13.27 (2.78)	0.003 (0.01)
**LVV**	5.08 (1.92)	5.24 (1.91)	18.29 (1.67)	11.08 (3.56)	0.0001 (0.01)
**RVV**	4.76 (3.78)	4.88 (3.75)	18.49 (1.63)	12.11 (3.41)	0.005 (0.01)
**HC Participants**					
**LAUD**	4.40 (1.36)	4.56 (1.33)	19.97 (1.89)	13.79 (3.20)	0.007 (0.02)
**RAUD**	4.01 (1.38)	4.21 (1.32)	19.66 (2.00)	14.21 (4.27)	0.008 (0.02)
**LSM**	3.97 (1.10)	4.26 (1.05)	18.46 (1.36)	14.93 (3.21)	0.003 (0.02)
**RSM**	3.71 (0.84)	4.02 (0.80)	18.00 (1.19)	16.47 (3.93)	0.004 (0.02)
**LVV**	4.25 (1.59)	4.45 (1.55)	18.63 (1.72)	13.63 (3.53)	0.002 (0.02)
**RVV**	3.61 (1.04)	3.82 (1.00)	18.72 (1.52)	15.22 (3.76)	0.006 (0.02)
***T-*tests**					
**LAUD**	-0.728 (.47)	-0.63 (.53)	-0.86 (.39)	-5.50 (<.000)	-1.87 (.06)
**RAUD**	2.00 (.05)	1.84 (.07)	-0.80 (.43)	-5.06 (<.000)	1.21 (.23)
**LSM**	1.20 (.24)	0.96 (.34)	-3.78 (.0003)	-4.33 (<.000)	-0.82 (.41)
**RSM**	2.99 (.004)	2.40 (.02)	-1.36 (.18)	-5.75 (<.000)	0.66 (.51)
**LVV**	2.40 (.02)	2.32 (.02)	-1.10 (.28)	-3.89 (.003)	-0.62 (.54)
**RVV**	1.83 (.07)	1.71 (.09)	-0.79 (.44)	-4.90 (<.000)	0.63 (.53)

*Note*. Values are presented as means (standard deviations). Full width at half max (FWHM) of water; Signal-to-noise ratio (SNR); Δδ_0_ = average frequency offset. Group differences in quality metrics was assessed using *t*-tests, shown as *t*-values (*p*-values).

### Statistical Analyses

Group differences in α-corrected GABA+ measurements for all voxels relative to water were analyzed using analysis of covariance (ANCOVA) including nuisance demographic variables of age, sex, education, race, and fit error. Because most prior studies examining GABA levels in MCI patients used creatine as a reference, additional analyses were performed with GABA+ referenced to creatine. These analyses could not control for tissue corrections in the same way as GABA referenced to water. Thus, to still correct for gray matter (GM) and white matter (WM) fractions of the voxels, GM and WM percentages were included in the ANCOVAs for GABA+ referenced to creatine (GABA+/Cr). All statistical analyses were conducted in *R* [37]. Analyses of group differences in tissue fractions are also included below (Figures 4 and 5) with full table results in the *Supplementary Material*. Additional analyses regarding group differences in glutamine/glutamate (Glx), relationships to cognition, and MCI subgroup differences are also provided in the *Supplementary Material.* All *a priori* hypotheses of group differences were tested using planned comparisons [38]. Therefore, no correction for multiple comparisons was applied.

We also modeled GABA in all voxels within a single statistical model that accounts for demographics and data quality (fit error). We refer to this as the full model in the results. Specifically, group differences in GABA+ measurements were analyzed using a linear mixed-effects model with group (MCI or HC), hemisphere (left or right), and data quality (fit error) included as fixed effects. We also included two covariates to account for the three different regions (visual, auditory, and motor) and these were effect-coded, using one of the regions as the reference. For example, when using the auditory region as reference, one covariate would have the value 1 for data in the visual regions, 0 for data in the sensorimotor regions, and -1 for data in the auditory regions, while the other covariate would have the value 1 for the sensorimotor regions, 0 for the visual regions, and -1 for the auditory regions. We ran models using each region as the reference region so that we could confirm parameter estimates and *p*-values for all three regions.

## RESULTS

The full model results for α-corrected GABA+ relative to water are shown in Table 3. In this full model, the MCI group exhibited significantly lower levels of GABA+ across all voxels when compared to healthy controls (*b* = -0.14, *p* < 0.001). Further, GABA+ levels differed by region, with the sensorimotor region (*b* = 0.24, *p* < 0.001) exhibited higher-than-global average levels of GABA+ while the auditory (*b* = -0.11, *p* < 0.001) and ventrovisual region (*b* = -0.35, *p* < 0.001) exhibited lower-than-global average levels of GABA+. Additionally, the left hemisphere voxels exhibited lower-than-global average levels of GABA+ (*b* = -0.05, *p* <0.001). Females exhibited slightly more GABA+/water than males (*b* = 0.09, *p* = 0.002) and model fit error of the GABA signal also was associated with overall GABA+ levels, in that those with higher fit error exhibited lower GABA+/water values (*b* = -0.05, *p* = <0.001). No other covariate reached statistical significance (e.g., age, education).

**Figure 2.**
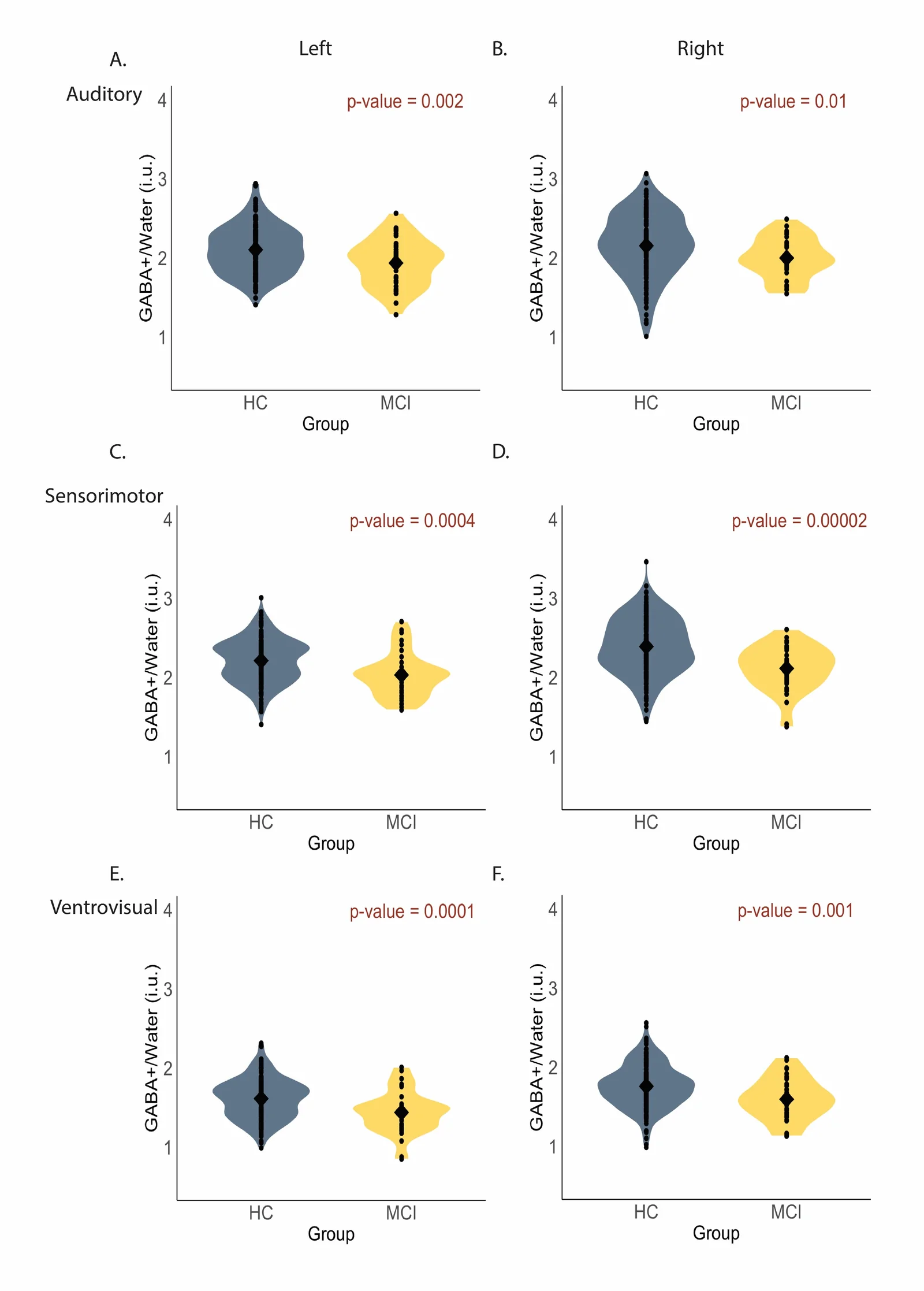
**α-corrected GABA+ level group differences**. α-corrected GABA+ levels referenced to water for left **(A)** and right **(B)** auditory voxels, left **(C)** and right **(D)** sensorimotor voxels, and left **(E)** and right **(F)** ventrovisual voxels. Fixed effect of group p-values are presented in red if significant and black if not significant. MCI (n=37) are in yellow and HC (n=163) are in blue.

Within-voxel analysis results for α-corrected GABA+ are shown in Figure 2 (full result tables are presented in *Supplementary Material*). The MCI group exhibited significantly lower levels of α-corrected GABA+ relative to water in all six brain regions: left auditory (*b* = -0.17, *p* = 0.002), right auditory (*b* = -0.10, *p* = 0.011), left sensorimotor (*b* = -0.15, *p* < 0.001), right sensorimotor (*b* = -0.27, *p* < 0.001), left ventral visual (*b* = -0.10, *p* = <0.001), and right ventral visual (*b* = -0.09, *p* = 0.001

**Table 3:** Full Model Results for α-corrected GABA+.

Variable	*b*	*p*-value
**Intercept**	2.216	**.000**
**Group**	-0.140	**.000**
**Age**	-0.001	.813
**Sex**	0.092	**.002**
**Education**	-0.005	.489
**Race**	-0.018	.547
**Fit Error**	-0.050	**.000**
**Hemisphere**	0.054	**.000**
**Sensorimotor**	0.244	**.000**
**Ventrovisual**	-0.352	**.000**
**Auditory**	-0.108	**.000**

*Note.* Significant *p*-values in bold.

The full model results for GABA+/Cr are shown in Table 4. In the full model, the MCI group exhibit significantly lower levels of GABA+/Cr levels across all voxels when compared to healthy controls (*b* = -0.004, *p =* 0.031). Additionally, GABA+/Cr levels differed by region, with the sensorimotor (*b* = 0.02, *p* < 0.001) and auditory (*b* = 0.01, *p* < 0.001) regions showing higher-than-global average levels of GABA+/Cr and the ventrovisual region (*b* = -0.01, *p* < 0.001) showing lower-than-average levels of GABA+/Cr. Model fit error of the GABA signal was associated with overall GABA+/Cr levels, in that those with higher fit error exhibited lower GABA+ values (*b* = -0.002, *p* = <0.001). All other covariates were not significant (e.g., GM, WM, fit error, age, sex, education).

**Figure 3.**
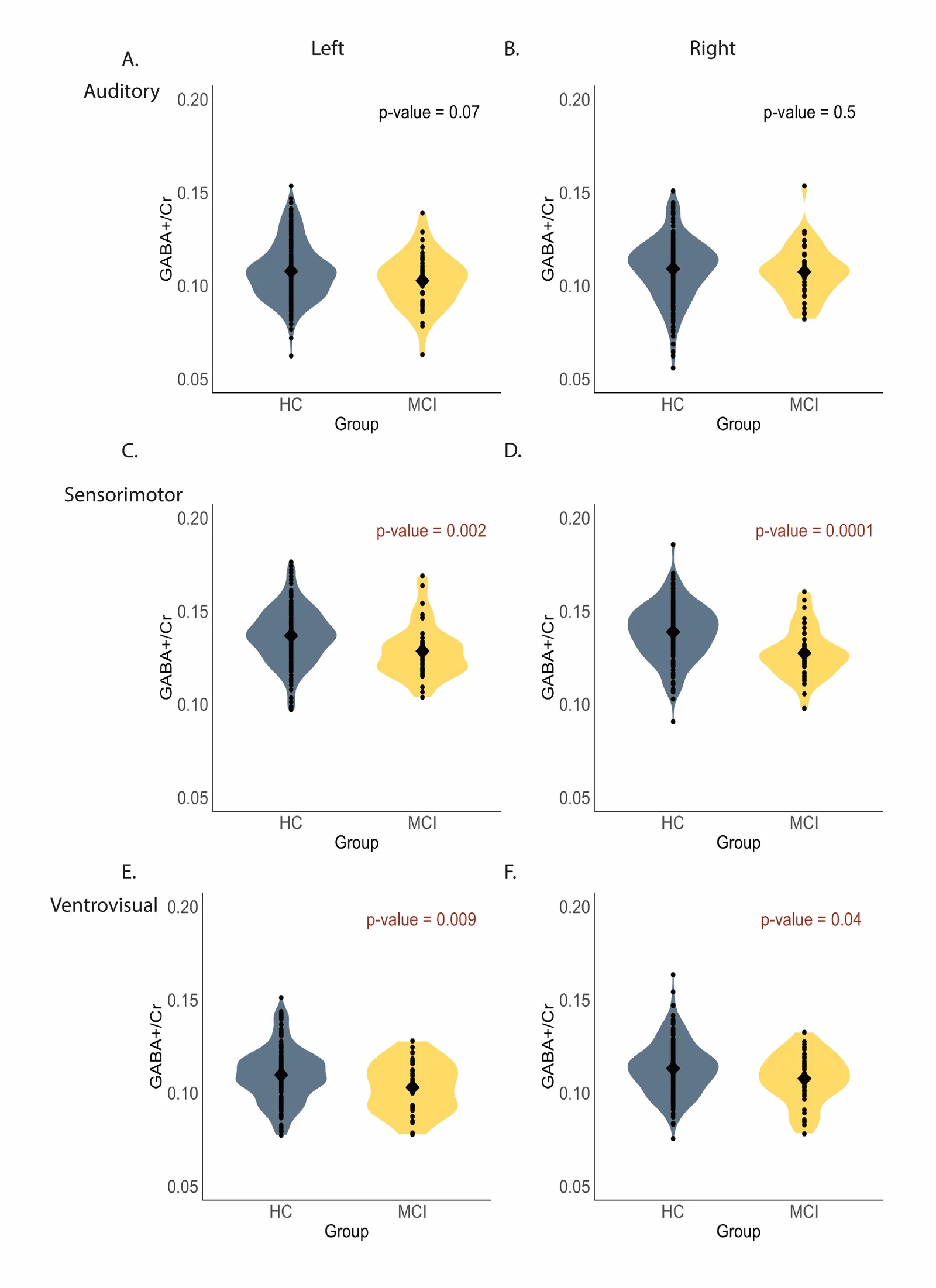
**GABA+/Cr levels group differences**. GABA+/Cr levels for left **(A)** and right **(B)** auditory voxels, left **(C)** and right **(D)** sensorimotor voxels, and left **(E)** and right **(F)** ventrovisual voxels. Fixed effect of group p-values are presented in red if significant and black if not significant. MCI (n=37) are in yellow and HC (n=163) are in blue.

**Table 4:** Full Model Results for GABA+/Cr.

Variable	*b*	*p*-value
**Intercept**	0.148	**.000**
**Group**	-0.004	**.031**
**Age**	0.000	.109
**Sex**	0.003	.070
**Education**	0.000	.929
**Race**	-0.003	.062
**Fit Error**	-0.002	**.000**
**Hemisphere**	0.001	**.015**
**Gray Matter (%)**	-0.006	.632
**White Matter (%)**	-0.012	.157
**Sensorimotor**	0.018	**.000**
**Ventrovisual**	-0.007	**.000**
**Auditory**	0.011	**.000**

*Note.* Significant *p*-values in bold.

Within-voxel analysis results for GABA+/Cr are shown in Figure 3 (full result tables presented in *Supplementary Material*). The MCI group exhibited significantly lower levels of GABA+/Cr in left sensorimotor (*b* = -0.004, *p* = 0.002), right sensorimotor (*b* = -0.01, *p* < 0.001), left ventral visual (*b* = -0.004, *p* = 0.009), and in right ventral visual voxels (*b* = -0.002, *p* = 0.041). There were no significant group differences for GABA+/Cr in left or right auditory voxels.

**Figure 4.**
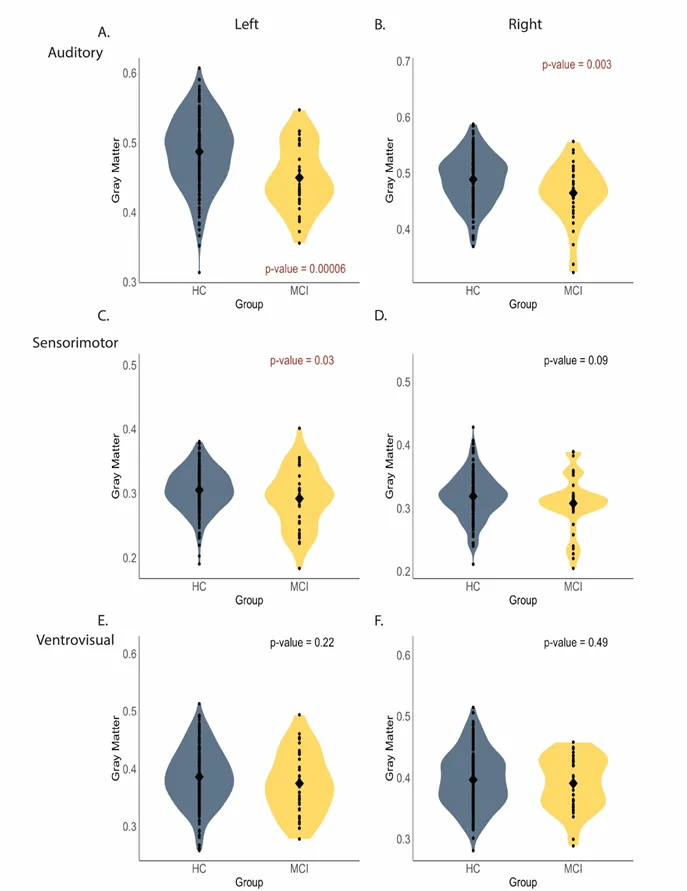
**Group gray matter fraction differences**. Group gray matter fraction differences for left **(A)** and right **(B)** auditory voxels, left **(C)** and right **(D)** sensorimotor voxels, and left **(E)** and right **(F)** ventrovisual voxels. Fixed effects of group p-values are presented in red if significant and black if not significant. MCI (n=37) are in yellow, and HC (n=163) are in blue. Within-voxel analysis results for white matter fractions are shown in Figure 5 (full result tables presented in *Supplementary Material*). The MCI group exhibited significantly lower levels of white matter fraction in the left sensorimotor voxel only (*b* = -0.034, *p* = 0.014).

Within-voxel analysis results for gray matter fractions are shown in Figure 4 (full result tables presented in *Supplementary Material*). The MCI group exhibited significantly lower levels of gray matter fraction in left auditory (*b* = -0.026, *p* < 0.000), right auditory (*b* = -0.01, *p* = 0.003), and left sensorimotor (*b* = -0.0005, *p* = 0.03) voxels. These differences underscore the importance of controlling for tissue fraction when comparing these two groups.

**Figure 5.**
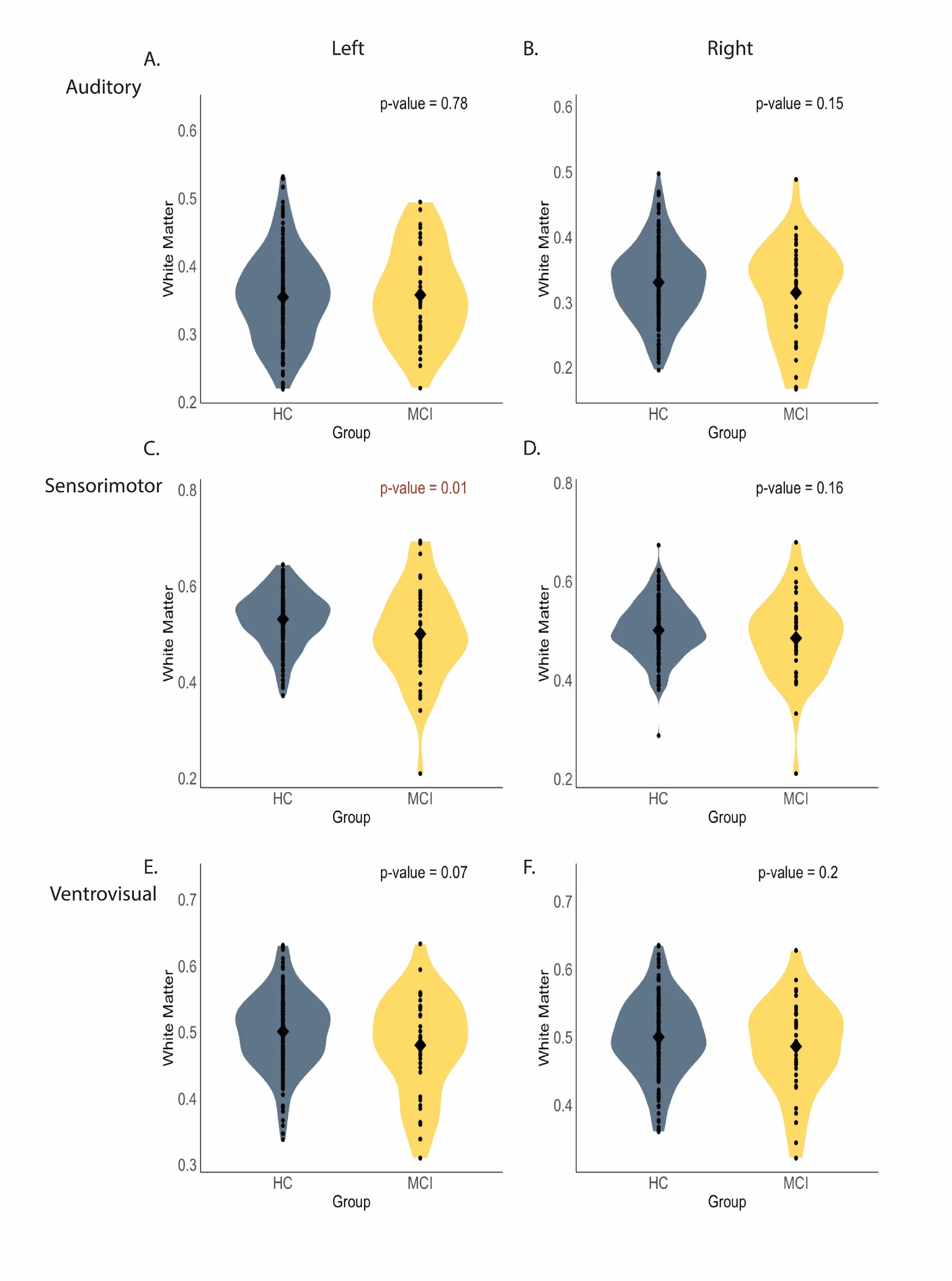
**Group white matter fraction differences**. Group gray matter fraction differences for left **(A)** and right **(B)** auditory voxels, left **(C)** and right **(D)** sensorimotor voxels, and left **(E)** and right **(F)** ventrovisual voxels. Fixed effects of group p-values are presented in red if significant and black if not significant. MCI (n=37) are in yellow, and HC (n=163) are in blue.

## DISCUSSION

The main aim of the current study was to investigate MCI-related differences in GABA levels in left and right visual, auditory, and sensorimotor cortex, where such differences have not been investigated before. This is also the first study to address knowledge gaps about the effect of tissue correction and reference molecule choice in MRS studies of pwMCI. The MCI group exhibited significantly lower levels of GABA compared to the healthy control group, and this result was robust to tissue correction whether GABA was referenced to water or creatine. When modeling GABA referenced to water within each voxel separately, the pwMCI showed significantly lower levels of GABA in all six regions even after correcting for tissue composition. We found a similar result in four of the six voxels when GABA was referenced to creatine (the exceptions were the left and right auditory voxels).

Findings from this study extend previous research that has observed significantly lower levels of GABA in the anterior and posterior cingulate cortex of pwMCI [15-17]. In particular, it demonstrates that pwMCI exhibit reduced GABA levels in additional brain regions that are not as well-researched in MCI (auditory cortex, sensorimotor cortex, and the ventrovisual cortex), suggesting that reductions in GABA may be a ubiquitous feature of the MCI brain.

The current findings are consistent with theories that hypothesize that age- and AD-related cognitive impairment are associated with synaptic dysfunction and associated neural hyperactivity. For example, the inhibition theory of aging postulates that age-related deficits in inhibitory processing play a major role in many of the cognitive impairments commonly observed in older adults [39-41]. Neuronal dysfunction and hyperactivity have also been observed to precede structural atrophy in Alzheimer’s disease and to be associated with cognitive impairment [5]. Increased activity in the frontal and temporal cortices has also been associated with poorer performance on memory tasks in Alzheimer’s disease [42].

GABA dysfunction has also been associated with age-related neural dedifferentiation, the finding that neural activation patterns elicited by different categories of sensory stimuli are less distinctive and more confusable in older vs. younger adults [43, 44]. In particular, individual differences in GABA levels have been found to be significantly associated with individual differences in neural dedifferentiation [44-48]. Furthermore, less distinctive neural representations have been associated with age-related behavioral impairments [49-52].

## Limitations

It is important to point out a number of limitations with the study. First, MRS voxels are relatively large (3cm^3^) and include cerebrospinal fluid and white matter in addition to grey matter itself. Furthermore, MRS does not distinguish between intra- and extracellular GABA and does not measure GABA activity but only GABA concentration [53]. We therefore cannot know whether GABA measured in this study is associated with synaptic activity. A limitation inherent to the current methods is the estimated GABA signal is known to be contaminated by signals associated with macromolecules [31, 54]. We therefore cannot know for sure whether it is the co-edited macromolecules that are different between the groups or GABA. Another limitation is that a thorough analysis with behavioral or cognitive assessments were not performed in this study. Thus, it is still not clear whether the observed MCI-related declines in GABA are directly associated with changes in behavior. Lastly, the study population is relatively homogenous and may not be fully representative of the broader population of pwMCI.

## Conclusion

In summary, we demonstrate that MCI patients have significantly lower levels of GABA compared to healthy controls in bilateral sensorimotor, auditory, and ventrovisual cortex. The observed MCI-related reductions in GABA levels were robust to tissue correction when referenced to water or creatine. These results provide support for theories that posit GABAergic dysfunction and neuronal hyperactivity as underlying factors in MCI/DAT-related functional decline.

## Supplementary Materials

The Supplementary data can be found online at: www.aginganddisease.org/EN/10.14336/AD.2025.0334.



## Data Availability

The code used to analyze the MRS data in this study is freely available as a toolbox and can be found here: https://markmikkelsen.github.io/Gannet-docs/index.html. Data is available upon request.
