# Supplementary material for: GABA Levels Are Significantly Reduced in the Visual, Motor, and Auditory Cortex of Patients with Mild Cognitive Impairment
Source: Aging Dis. 2025 Jun 26;17(4):2241–52. doi: 10.14336/AD.2025.0334 (PMC13256622; doi:10.14336/AD.2025.0334)
Supplement: Supplementary file 1 [file AD-17-4-2241-s.pdf]

SUPPLEMENTARY DATA

GABA Levels Are Significantly Reduced in the Visual, Motor, and Auditory Cortex of Patients with Mild Cognitive Impairment

Mark D. Zuppichini, Abbey M. Hamlin, Quan Zhou, Esther Kim, Kayla Wyatt, Noah Reardon[2], Benjamin M. Hampstead, Thad A. Polk

© 2025. Zuppichini MD. et al. Published online at http://www.aginganddisease.org/EN/10.14336/AD.2025.0334

SUPPLEMENTARY DATA

Supplementary Material

Results

Within-voxel analysis results for **α**-corrected GABA+ are shown in Figure 2 (main manuscript; full result tables are presented below). The MCI group exhibited significantly lower levels of **α**-corrected GABA+ relative to water in all six brain regions: left auditory (b = -0.17, p = .002), right auditory (b = -0.10, p = .011), left sensorimotor (b = -0.15, p < .001), right sensorimotor (b = -0.27, p < .001), left ventrovisual (b = -0.10, p < .001), and right ventral visual (b = -0.09, p = .001). Demographic and measure of fit variables were also significantly associated with GABA in several of the voxels (significant p-values bolded).

Supplementary Table 1. Left Auditory Voxel ATCG-GABA Model Results

Variable	*b*	*p*-value
Group	-0.174	**.002**
Age	0.002	.993
Sex	0.095	**.004**
Education	-0.004	.906
Race	0.002	.861
Fit Error	-0.051	**.002**

Note. Significant *p*-values in bold.

Supplementary Table 2. Right Auditory Voxel ATCG-GABA Model Results

Variable	*b*	*p*-value
Group	-0.095	**.011**
Age	-0.001	.174
Sex	0.018	.107
Education	-0.023	.070
Race	-0.007	.785
Fit Error	-0.144	**.000**

Note. Significant *p*-values in bold.

Supplementary Table 3. Left Sensorimotor Voxel ATCG-GABA Model Results

Variable	*b*	*p*-value
Group	-0.154	**.000**
Age	0.004	.419
Sex	0.106	**.015**
Education	0.003	.521
Race	-0.084	**.034**
Fit Error	-0.033	.077

Note. Significant *p*-values in bold.

© 2025. Zuppichini MD. et al. Published online at http://www.aginganddisease.org/EN/10.14336/AD.2025.0334

Supplementary Table 4. Right Sensorimotor Voxel ATCG-GABA Model Results

Variable	*b*	*p*-value
Group	-0.272	**.000**
Age	0.010	.211
Sex	0.079	**.024**
Education	-0.010	.337
Race	-0.009	.852
Fit Error	-0.093	**.003**

Note. Significant *p*-values in bold.

Supplementary Table 5. Left Ventrovisual Voxel ATCG-GABA Model Results

Variable	*b*	*p*-value
Group	-0.102	**.000**
Age	-0.006	**.025**
Sex	0.073	**.001**
Education	-0.008	.398
Race	0.043	.184
Fit Error	-0.066	**.000**

Note. Significant *p*-values in bold.

Supplementary Table 6. Right Ventrovisual Voxel ATCG-GABA Model Results

Variable	*b*	*p*-value
Group	-0.094	**.001**
Age	-0.004	.215
Sex	0.120	**.003**
Education	0.007	.442
Race	-0.088	**.034**
Fit Error	-0.017	.102

Note. Significant *p*-values in bold.

Within-voxel analysis results for GABA+/Cr are shown in Figure 3 (main manuscript; full result tables are presented below). The MCI group exhibited significantly lower levels of GABA+/Cr in left sensorimotor (b = -0.004, p = .002), right sensorimotor (b = -0.011, p < .001), left ventral visual (b = -0.004, p = .009), and right ventral visual (b = -0.002, p = .041) voxels. There were no significant group differences for GABA+/Cr in left or right auditory voxels. Demographic and measure of fit variables were also significantly associated with GABA in several of the voxels (significant *p*-values bolded).

© 2025. Zuppichini MD. et al. Published online at http://www.aginganddisease.org/EN/10.14336/AD.2025.0334

Supplementary Table 7. Left Auditory GABA+/Cr Model Results

Variable	*b*	*p*-value
Group	-0.004	.065
Age	0.000	.254
Sex	0.006	**.013**
Education	0.000	.489
Race	-0.002	.399
Fit Error	-0.002	**.004**
Gray Matter (%)	-0.016	.724
White Matter (%)	-0.028	.229

Note. Significant *p*-values in bold.

Supplementary Table 8. Right Auditory GABA+/Cr Model Results

Variable	*b*	*p*-value
Group	0.000	.462
Age	0.000	**.028**
Sex	0.001	.436
Education	-0.001	.374
Race	-0.001	.529
Fit Error	-0.004	**.000**
Gray Matter (%)	-0.008	.677
White Matter (%)	-0.030	.136

Note. Significant *p*-values in bold.

Supplementary Table 9. Left Sensorimotor GABA+/Cr Model Results

Variable	*b*	*p*-value
Group	-0.004	**.002**
Age	0.000	.908
Sex	0.002	.771
Education	0.000	.486
Race	-0.009	**.000**
Fit Error	-0.002	.283
Gray Matter (%)	0.014	.916
White Matter (%)	0.033	**.010**

Note. Significant *p*-values in bold.

Supplementary Table 10. Right Sensorimotor GABA+/Cr Model Results

Variable	*b*	*p*-value
Group	-0.011	**.000**

Age	0.000	.347
Sex	0.000	.871
Education	0.000	.458
Race	-0.004	.147
Fit Error	-0.004	**.010**
Gray Matter (%)	-0.032	.490
White Matter (%)	-0.012	.535

Note. Significant *p*-values in bold.

Supplementary Table 11. Left Ventrovisual GABA+/Cr Model Results

Variable	*b*	*p*-value
Group	-0.004	**.009**
Age	0.000	**.049**
Sex	0.001	.475
Education	0.000	.481
Race	0.000	.793
Fit Error	-0.003	**.000**
Gray Matter (%)	-0.048	.474
White Matter (%)	-0.051	**.010**

Note. Significant *p*-values in bold.

Supplementary Table 12. Right Ventrovisual GABA+/Cr Model Results

Variable	*b*	*p*-value
Group	-0.002	**.041**
Age	0.000	.228
Sex	0.004	.208
Education	0.001	.259
Race	-0.005	**.009**
Fit Error	0.000	.511
Gray Matter (%)	-0.017	.573
White Matter (%)	-0.006	.782

Note. Significant *p*-values in bold.

Additional Results

Similar to the main analyses, group differences in tissue-corrected Glx estimates for all voxels relative to water were analyzed using analysis of covariance (ANCOVA) including nuisance demographic variables of age, sex, education, race, and fit error. Results from ANCOVAs are illustrated in Supplemental Figure 1. The MCI group exhibited significantly lower tissue-corrected Glx estimates in left auditory (b = -0.68, p = .013), left ventral visual (b = -0.45, p = .010), and right ventral visual (b = -0.68, p = .034) voxels. Full results from this exploratory analysis are now included in the supplementary material.

© 2025. Zuppichini MD. et al. Published online at http://www.aginganddisease.org/EN/10.14336/AD.2025.0334

SUPPLEMENTARY DATA

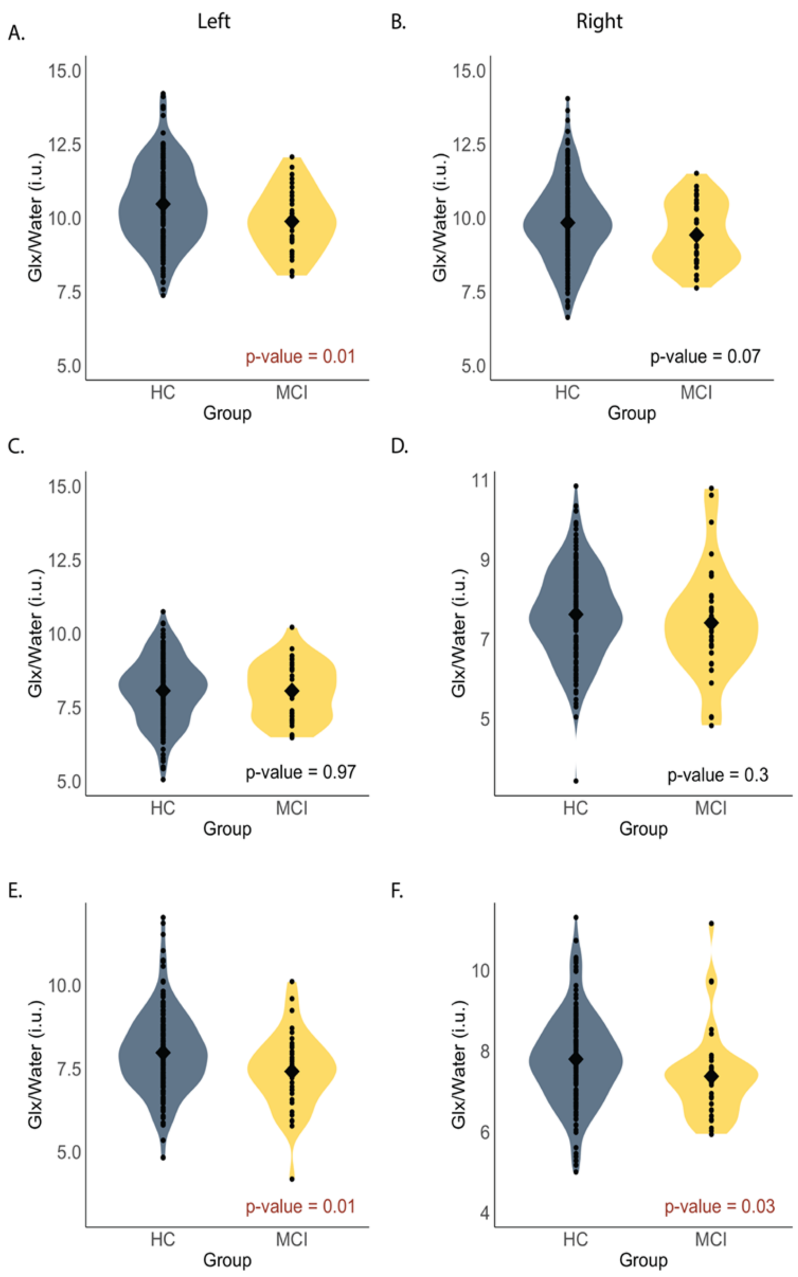

Supplementary Figure 1. Tissue-corrected Glx estimates referenced to water for left (A) and right (B) auditory voxels, left (C) and right (D) sensorimotor voxels, and left (E) and right (F) ventrovisual voxels. Fixed effect of group p-values are presented in red if significant and black if not significant. MCI (n=37) are in yellow and HC (n=163) are in blue.

We were also interested in investigating the relationship between cognitive measures and GABA estimates. At the moment, however, our sample size is significantly underpowered to try to relate GABA to other variables, especially behavioral measures. Nevertheless, we did try an exploratory linear regression analysis assessing the relationship between **α**-corrected GABA+ estimates and MoCA scores within the MCI group (Supplemental Figure 2 below). **α**-corrected GABA+ estimates in the left ventral

© 2025. Zuppichini MD. et al. Published online at http://www.aginganddisease.org/EN/10.14336/AD.2025.0334

visual were significantly associated with MoCA scores in the MCI group ($R^2 = 0.24$, $p = .002$), but this relationship was not significant in any other voxel. We hope to conduct such a study on a larger dataset in the future so we chose not to include these results here, but we certainly could if the reviewers think it would improve the paper.

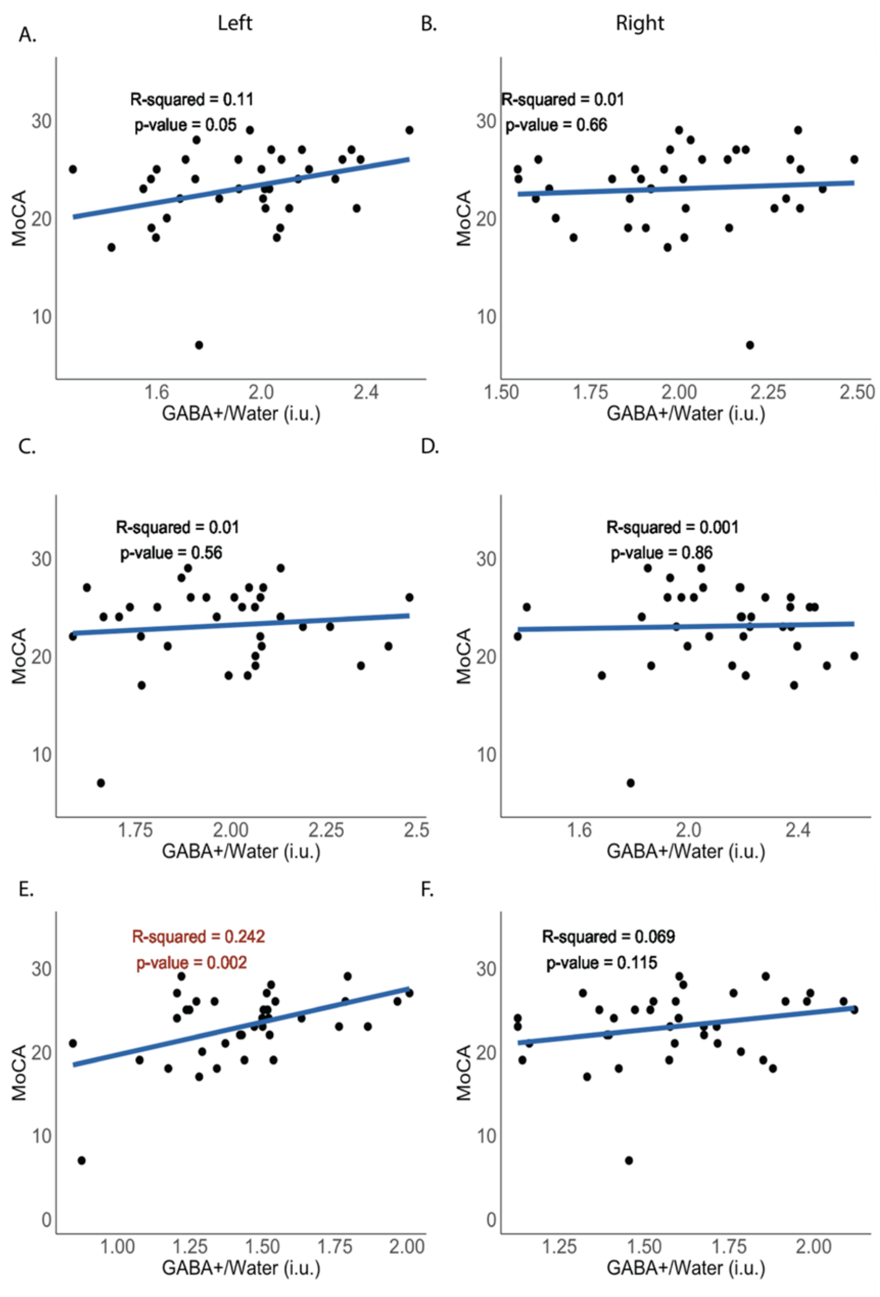

Supplementary Figure 2. Linear regression results for the relationship between MoCA scores and GABA+/water in left (A) and right (B) auditory voxels, left (C) and right (D) sensorimotor voxels, and left (E) and right (F) ventrovisual voxels within the MCI group.

SUPPLEMENTARY DATA

We were also interested in MCI subgroup differences. However, there are only 27 amnestic MCI (aMCI) and 10 non-amnestic MCI (naMCI) in our sample. We did try performing analyses similar to the analyses in the main text comparing these subgroups using ANCOVAs and including nuisance demographic variables of age, sex, education, race, and fit error. Results are shown in Supplemental Figure 3. There were no significant differences in **α**-corrected GABA+ estimates between MCI subgroups in any voxel. We decided not to include these results in the revision, but could do so if the reviewers feel strongly that we should.

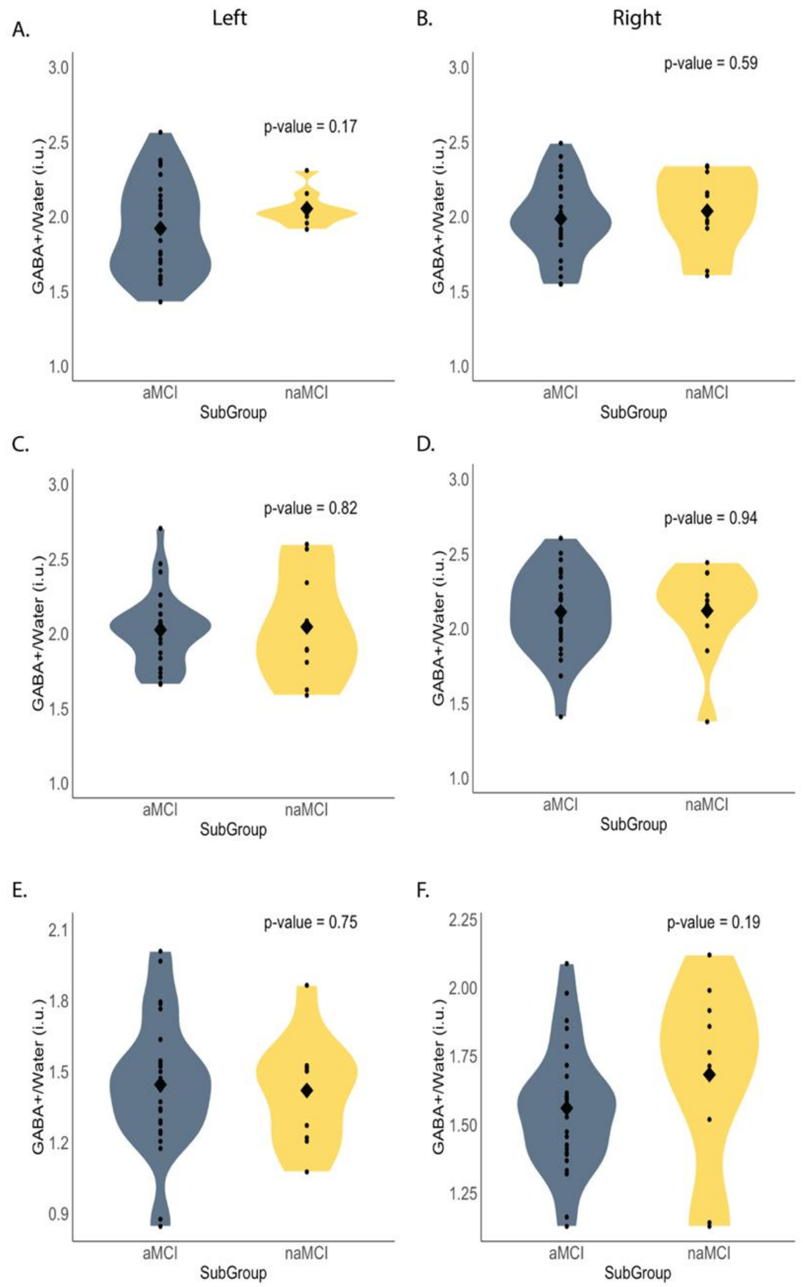

Supplementary Figure 3. MCI subgroup differences in GABA+/water for left (A) and right (B) auditory voxels, left (C) and right (D) sensorimotor voxels, and left (E) and right (F) ventrovisual

© 2025. Zuppichini MD. et al. Published online at http://www.aginganddisease.org/EN/10.14336/AD.2025.0334

voxels. Fixed effect of group p-values are presented in red if significant and black if not significant. Amnestic MCI (aMCI; n=17) are in blue and non-amnestic MCI (naMCI; n=10) are in yellow.

Tissue fractions were also investigated for group differences. Results from ANCOVAs including nuisance variables of age, sex, education, and race are presented in Supplemental Figure 4 & 5. The MCI group exhibited significantly lower gray matter fraction in the left auditory (b = -0.03, p < .001), right auditory (b = -0.01, p = .003), and left sensorimotor (b = -0.001, p = .033) voxels compared to the HC group. No other voxels exhibited significant group differences in gray matter fraction (Supplemental Figure 4). Furthermore, the MCI group exhibited significantly lower white matter fraction in the left sensorimotor (b = -0.03, p = .014) voxel (but in no other voxels) compared to the HC group (Supplemental Figure 5). Figures and tables with full model results are now included in the supplemental material.

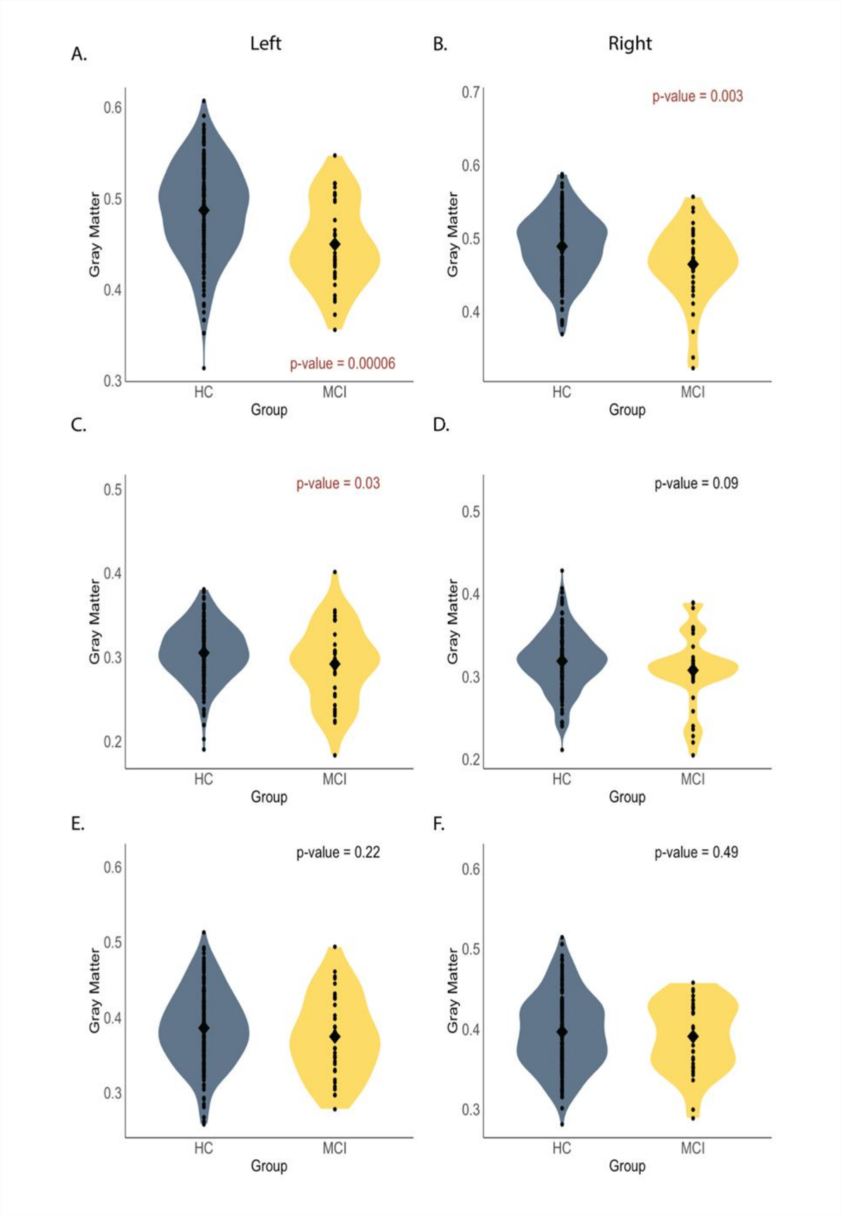

© 2025. Zuppichini MD. et al. Published online at http://www.aginganddisease.org/EN/10.14336/AD.2025.0334

SUPPLEMENTARY DATA

Supplementary Figure 4. Gray Matter fractions for left (A) and right (B) auditory voxels, left (C) and right (D) sensorimotor voxels, and left (E) and right (F) ventrovisual voxels. Fixed effect of group p-values are presented in red if significant and black if not significant. MCI (n=37) are in yellow and HC (n=163) are in blue.

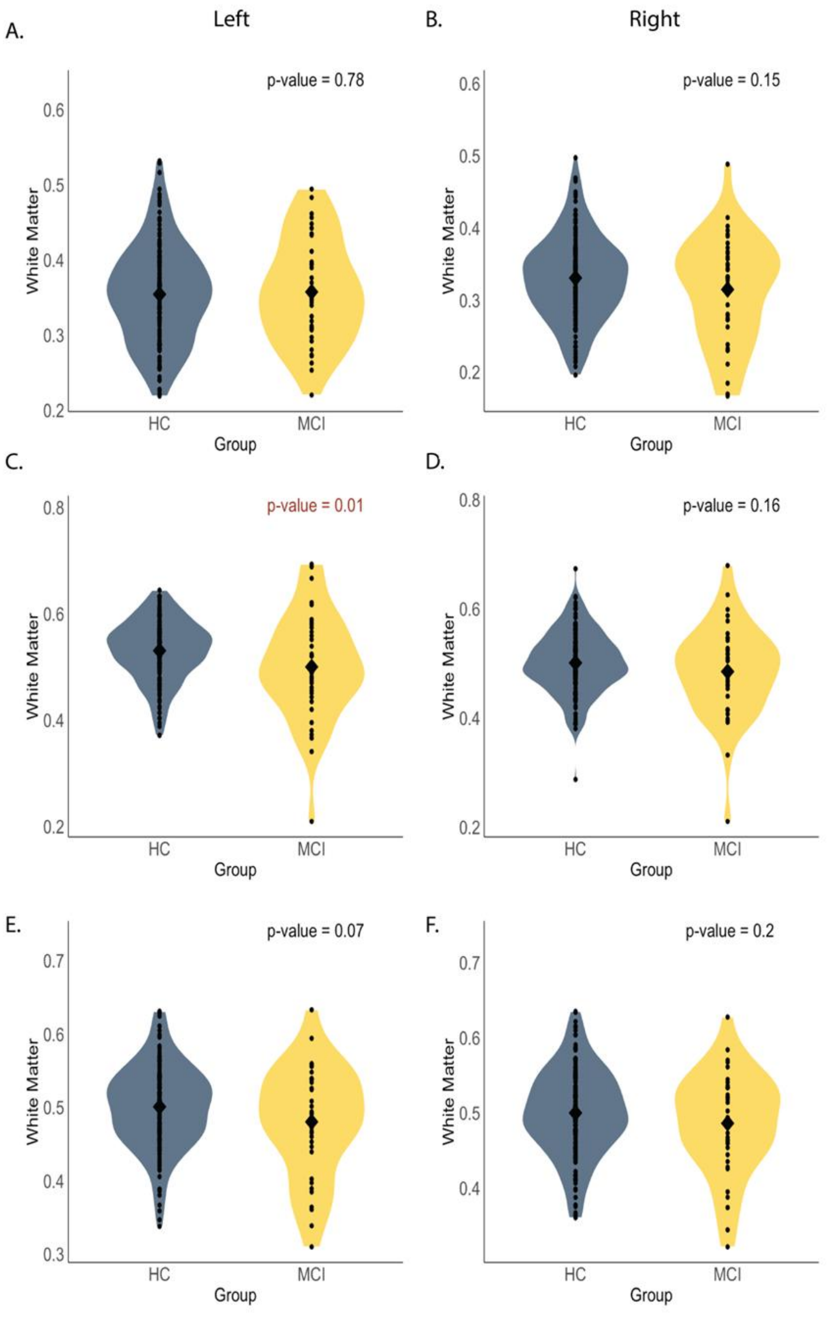

Supplementary Figure 5. White Matter fractions for left (A) and right (B) auditory voxels, left (C) and right (D) sensorimotor voxels, and left (E) and right (F) ventrovisual voxels. Fixed effect of group p-values are presented in red if significant and black if not significant. MCI (n=37) are in yellow and HC (n=163) are in blue.

© 2025. Zuppichini MD. et al. Published online at http://www.aginganddisease.org/EN/10.14336/AD.2025.0334
